# Keeping children safe at home: protocol for a case–control study of modifiable risk factors for scalds

**DOI:** 10.1136/injuryprev-2014-041255

**Published:** 2014-05-19

**Authors:** P Wynn, J Stewart, A Kumar, R Clacy, F Coffey, N Cooper, C Coupland, T Deave, M Hayes, E McColl, R Reading, A Sutton, M Watson, D Kendrick

**Affiliations:** 1 Division of Primary Care, School of Medicine, Nottingham, UK; 2 School of Health Sciences, University of Nottingham, Queen's Medical Centre, Nottingham, UK; 3 Nottingham University Hospitals NHS Trust, Queen's Medical Centre Campus, Nottingham, UK; 4 Department of Health Sciences, University of Leicester, Leicester, UK; 5 Centre for Child & Adolescent Health, Health and Life Sciences, University of the West of England, Bristol, UK; 6 Child Accident Prevention Trust, Canterbury Court (1.09), 1 - 3 Brixton Road, London, UK; 7 Great North Children's Hospital, Research Unit Level 2, New Victoria Wing, Royal Victoria Infirmary, Newcastle upon Tyne, UK; 8 Norfolk and Norwich University Hospitals NHS Foundation Trust, Norwich, UK

## Abstract

**Background:**

Scalds are one of the most common forms of thermal injury in young children worldwide. Childhood scald injuries, which mostly occur in the home, result in substantial health service use and considerable morbidity and mortality. There is little research on effective interventions to prevent scald injuries in young children.

**Objectives:**

To determine the relationship between a range of modifiable risk factors for medically attended scalds in children under the age of 5 years.

**Design:**

A multicentre case-control study in UK hospitals and minor injury units with parallel home observation to validate parental reported exposures. Cases will be 0–4 years old with a medically attended scald injury which occurred in their home or garden, matched on gender and age with community controls. An additional control group will comprise unmatched hospital controls drawn from children aged 0–4 years attending the same hospitals and minor injury units for other types of injury. Conditional logistic regression will be used for the analysis of cases and matched controls, and unconditional logistic regression for the analysis of cases and unmatched controls to estimate ORs and 95% CI, adjusted and unadjusted for confounding variables.

**Main exposure measures:**

Use of safety equipment and safety practices for scald prevention and scald hazards.

**Discussion:**

This large case-control study will investigate modifiable risk factors for scalds injuries, adjust for potential confounders and validate measures of exposure. Its findings will enhance the evidence base for prevention of scalds injuries in young children.

## Introduction

Burn injuries in children under 10 years of age are a major cause of death internationally, and are the fifth most common cause of non-fatal childhood injuries.^1^ In those under 5 years, scalds are the leading cause of thermal injuries in high, middle and low income countries.^2–14^ Scalds accounted for 62% of hospital admissions for thermal injuries in children under 5 years old in the USA between 2003 and 2012,^15^ and 63% of such admissions in the same age group in England in 2012–2013.^16^ In 2012–2013 over 3500 children aged 0–14 years were admitted to hospital in England with a thermal injury, of which the majority (61%) were scalds.^16^ Most scalds in childhood occur at home^12^ ^13^ ^17^ and are most commonly caused by hot liquids from kettles, baths, cups or mugs.^17^^–^19

Paediatric scald injuries impose a heavy financial burden on the state. More severe scalds may require intensive care, skin grafts and months or years of rehabilitation.^2^ Studies from England suggest the average cost of treating an uncomplicated minor (<10% total body surface area) paediatric scald in 2002–2003 was £1850^20^ while the average cost of acute inpatient treatment of a ‘major scald’ (30–40% total body surface area) in 2007–2009 in a paediatric burns unit can be as high as £55 000.^21^ Socioeconomic disadvantage increases the risk of scalds. Recent research from the UK demonstrates that children living in the most disadvantaged areas have an 82% higher odds of a medically attended scald injury than those in the most affluent areas.^22^ This pattern is repeated globally, with socioeconomic differentials between and within countries.^12^ ^13^ ^23^

Systematic reviews and recent meta-analyses have consistently demonstrated that interventions to promote thermal safety in the home can increase safety behaviour and the use of safety equipment to reduce the risk of thermal injuries.^24–31^ However, there remains little evidence that they reduce the actual incidence of thermal injuries due to a lack of adequately powered randomised controlled trials or high quality observational studies. Rigorous case–control studies have often provided the best evidence for effective interventions in injury prevention.^32–34^ There are few case–control studies investigating prevention practices in the homes of scalded children.^23^ ^35–37^ One Canadian multicentre study, exploring risk and protective factors for a range of childhood injuries requiring emergency department (ED) attendance in children under the age of 8 years, failed to find any significant associations between any scald prevention practices and ED attendance.^35^ A second study of children of all ages attending EDs in two hospitals in Greece with a thermal injury found a 40% reduction in the odds of injury with a one unit increase in a burn avoidance index which measured four scald prevention behaviours.^36^ Another study, of children aged 0–5 years attending an Iraqi hospital with an acute burn injury found a one unit increase in the number of thermal hazards increased the odds of a thermal injury by 32%.^23^ Finally a study of children aged 0–4 years attending EDs in 14 Dutch hospitals found that using a vacuum flask rather than cups or mugs, to store hot drinks halved the odds of ED attendance for a thermal injury.^37^ These studies were limited by small sample sizes,^36^ ^37^ the use of hospital controls,^23^ ^35^ ^36^ the use of unvalidated parental reports to measure exposures,^23^ ^36^ ^37^ case definitions covering a wide range of injury mechanisms^35^ and potential bias from unmeasured confounders.^23^ ^35–37^

The aim of the case–control study described in this protocol is to examine the relationship between modifiable risk factors and scald injuries in young children. Community and hospital controls will be used and a range of confounding factors will be measured and adjusted for. Parental reported exposures will be validated by home observations in a sample of cases and controls.

## Methods

### Objectives

The objective of this case–control study is to determine the relationship between a range of modifiable risk factors and medically attended scalds in children under the age of 5 years.

### Study design

A multicentre case–control study will be carried out within hospitals and minor injury units (MIUs) in Nottingham, Bristol, Derby, Gateshead, Great Yarmouth, Newcastle upon Tyne, Norwich and Lincoln, UK. This is one of a series of case-control studies running concurrently in these study centres which employ identical methods and aim to identify modifiable risk factors for different injury mechanisms. The published protocol for the falls case–control study describes the methods in more detail.^38^

### Definition of cases and controls

Cases will be defined as children under the age of 5 years who received medical attention for a scald injury which occurred in their home or garden, defined as the address at which they were registered with a family doctor or general practitioner (GP). Scald injuries are defined as ‘thermal injuries caused by hot liquids or steam’. Medical attention is defined as an admission to hospital or attendance at an ED or MIU. Children are not eligible to take part if the injury was intentional or suspected to be intentional or if the child lives in residential care. Fatal injuries will not be included to avoid parental distress. Cases are not eligible to be included as a control in the study at any time after being recruited as a case.

Controls are defined as children under the age of 5 years who did not receive medical attention for a scald injury on the date of the case's medical attendance. Children living in residential care are not eligible to participate in the study. Controls are eligible for recruitment to the study as a case or as a further control if they are recruited at least 12 months after their first recruitment. Controls will not be recruited more than twice to the study.

### Recruitment of cases and controls

Participants will be recruited during or after their hospital admission or attendance at the ED or MIU. The initial approach to potential participants will be made by clinical staff, followed by contact from researchers where agreement for this is given. Researchers will contact potential participants during their medical attendance, or by telephone or postal invitation within 72 h of ED or MIU attendance. Postal and telephone reminders will be used if no response is received within 2 weeks. Participants will be given a £5 voucher for local stores to thank them for participation in the study.

Controls will be recruited from the community (matched community controls) and from the hospital (unmatched hospital controls). Community controls will be identified from the GP practice where the case is registered, and matched with the case on age (within 4 months) and gender. If the GP practice where the case is registered does not consent to take part, the GP practice closest to the case's GP practice will be asked to recruit controls. GPs or Primary Care Trust staff will invite, by post, parents or guardians (hereafter referred to as parents) of 10 children for each recruited case to take part in the study. One reminder will be sent to non-responders (controls and cases) if they have not replied within 2 weeks of initial mailout. Hospital controls will comprise children already recruited as a case to one of the other ongoing case–control studies, that is, these will be children aged 0–4 years attending the ED or MIU with another injury mechanism (fall on the same level, fall from furniture, staircase fall or poisoning).

### Definition of exposures and confounding variables

Exposures relating to scalds will be categorised into safety (and other potentially risk reducing) equipment use, safety behaviours and home hazards (see table 1). Cases will be asked about exposures relating to the 24 h (or 1 week for less frequent behaviours) before the scald injury. Controls will be asked about exposures for the 24 h (or 1 week for less frequent behaviours) prior to completing the questionnaire. A range of potential confounding factors will be measured as shown in table 1.

**Table 1:** Definition of exposures and confounding variables

*Exposure: Safety and other potentially risk reducing equipment use* Use of safety gates Kettles with curly or short cables Play pens (or travel cots) Stationary activity centres *Exposure: Safety behaviours* Not drinking hot drinks while holding a child Not passing hot drinks over a child Keeping hot drinks out of reach of children Storing kettles at back of work tops Use of back rings on cooker Turning saucepan handles away from edge of cooker Not using tablecloths Knowledge of hot tap water/thermostat temperature Using cold water first when running a bath Measuring bath water temperature Not leaving child without an adult in the bath or bathroom Not having children running baths Teaching children safety rules about hot liquids *Exposure: Home hazards* Has baby walker	*Potential confounders: sociodemographic* Age Gender Ethnic group Family size and structure Housing tenure Receipt of state-provided means-tested benefits Single parenthood Adult unemployment in the household Overcrowding Deprivation (measured using the Index of Multiple Deprivation^39^) Distance of residence from hospital Use of out-of-home childcare *Potential Confounders**:** child and parent measures for health and behaviour* Child behaviour (infant, early child and child behaviour questionnaires)^40–42^ Child health status (VAS^43^; PedsQL^44^ ^45^) Long-term health conditions Parental mental health (Hospital Anxiety and Depression Scale)^46^ Parenting daily hassles^47^ ^48^ Parental perception of child's ability to reach hot liquids (a series of questions on climbing, reaching, turning on taps, ability to open safety gates)

PedsQL, pediatric quality of life inventory; VAS, visual analogue scale.

### Measurement of exposures and confounding variables

Exposures and confounding factors will be measured using parent-completed questionnaires, specific to the age of the child (0–12 months, 13–36 months and 37–59 months). The development, piloting and validation of the questionnaires are described in the protocol for the falls case–control study.^38^

### Analysis

The analysis of the validation of exposures has been described in the falls case–control study protocol.^38^ Relationships between exposures, potential confounding variables and scalds will be explored using causal diagrams. These will be used to identify the minimal set of potential confounding factors to adjust for when estimating ORs. Exposure and confounding variables will be described using frequencies and percentages for categorical variables and means (and SDs) or medians (and IQRs) for continuous variables as appropriate.

Conditional logistic regression will be used for the analysis of cases and matched controls to estimate ORs and 95% CIs, unadjusted and adjusted for confounding variables. All models will a priori adjust for deprivation and distance from hospital. Unconditional logistic regression will be used for the analysis of cases and hospital controls. All models will a priori be adjusted for age, gender, deprivation and distance from hospital, in addition to other potential confounding variables, as described for the matched analysis. Effect modification will be investigated by adding interaction terms to models and where present (p<0.01), stratified results will be reported. Results will also be stratified by the type of service used (child treated in ED or admitted to hospital vs no treatment required) as a proxy for injury severity.

### Sample size

Based on data on the prevalence of exposures and the amount of missing data obtained from analysis of the first 428 controls recruited across all the ongoing case–control studies, 259 cases and 1036 matched controls are required to detect an OR of 0.64 (equivalent to an OR of 1.56 for a risk factor), with 80% power, a 5% significance level, a correlation between matched cases and controls of 0.1 and an average of four controls per case.

### Ethics committee and regulatory approvals

Ethical approval was provided by the Nottingham 1 Ethics Committee (reference number: 09/H0407/14). Approval has been obtained from National Health Service research and development departments providing research governance to participating hospitals and MIUs.

## Discussion

This large, multicentre case–control study will investigate modifiable risk factors for scald injuries in the homes of children under the age of 5 years. The study will attempt to address some of the limitations of previous studies and will use multiple methods to minimise a range of biases. Cases and community controls will be matched on age and gender, and hospital and community controls will be used. A wide range of exposures will be measured, using, where possible, previously validated questions to help minimise misclassification bias. In addition, a concurrent study validating self-reported exposures will enable quantification of under-reporting or over-reporting of exposures by cases and controls.^38^ Further efforts to reduce misclassification bias include restricting measures of exposure to a short time period (maximum of 1 week) prior to the injury for cases, and prior to questionnaire completion for the controls. Data will be collected on a wide range of confounding factors using validated tools wherever possible. The findings from this study should provide evidence to inform scald prevention policy and practice, with implications for child health surveillance programmes and home safety equipment schemes. Our findings should also inform the advice provided during home safety assessments and other child health promotion contacts.
